# High-intensity interval training in children and adolescents with special educational needs: a systematic review and narrative synthesis

**DOI:** 10.1186/s12966-023-01421-5

**Published:** 2023-02-09

**Authors:** Eric Tsz-Chun Poon, Waris Wongpipit, Fenghua Sun, Andy Choi-Yeung Tse, Cindy Hui-Ping Sit

**Affiliations:** 1 Department of Health and Physical Education, The Education University of Hong Kong, Taipo, Hong Kong; 2 Division of Health and Physical Education, Faculty of Education, Chulalongkorn University, Bangkok, Thailand; 3 Thailand Physical Activity Knowledge Development Centre, Institute for Population and Social Research, Mahidol University, Nakhon Pathom, Thailand; 4 Department of Sports Science and Physical Education, The Chinese University of Hong Kong, Shatin, Hong Kong

**Keywords:** HIIT, Interval exercise, Young people, Disabilities, Public health

## Abstract

**Background:**

High-intensity interval training (HIIT) has been promoted as a time-efficient exercise strategy to improve health and fitness in children and adolescents. However, there remains little consensus in the literature regarding its efficacy in children and adolescents with special educational needs (SEN). This study aimed to examine HIIT as a means of improving key health and fitness parameters in children and adolescents with SEN.

**Methods:**

A systematic search was conducted on eight databases (MEDLINE, Embase, SPORTDiscus, Web of Science, Scopus, PsycINFO, CINAHL, and Cochrane Library). Studies were eligible if they 1) included an HIIT protocol, 2) examined parameters related to both physical and mental aspects of health and fitness, and 3) examined children and adolescents with SEN aged 5–17 years.

**Results:**

Of the 1727 studies yielded by the database search, 13 (453 participants) were included and reviewed. We found that HIIT generally improved body composition, physical fitness, and cardiometabolic risk biomarkers across a spectrum of SEN (e.g., attention deficit hyperactivity disorder, cerebral palsy, developmental coordination disorder, and mental illness). Improvements in mental health and cognitive performance following HIIT have also been observed.

**Conclusion:**

This review provides up-to-date evidence for HIIT as a viable exercise strategy for children and adolescents with SEN. Further research investigating the benefits of HIIT in a wider range of SEN populations is warranted.

**Trial registration:**

This study was registered in the International Prospective Register of Systematic Review (PROSPERO; registration number CRD42022352696).

**Supplementary Information:**

The online version contains supplementary material available at 10.1186/s12966-023-01421-5.

## Introduction

Physical inactivity is a serious global health problem, and its association with non-communicable diseases, including cardiovascular diseases, obesity, type 2 diabetes mellitus, cancer, and premature mortality, is well documented [1]. The current World Health Organization guidelines on physical activity (PA) recommend a minimum of 60 min/day of moderate-to-vigorous-intensity aerobic PA for children and adolescents, including those living with disabilities [2, 3]. Although regular PA offers benefits for physical and mental well-being [4], children and adolescents with special educational needs (SEN) are considerably less physically active, tend to engage more in sedentary pursuits [5–8], and are at a higher risk for obesity [9] than their typically developing peers. While SEN can cover a range of needs, including physical or mental disabilities and cognition or educational impairments [10], it appears that children and adolescents with SEN, regardless of the type, face some common barriers (e.g., lack of knowledge and skills, inadequate facilities, and cost) when engaging in PA [11]. Furthermore, children and adolescents with SEN are more likely to develop mental health problems, such as anxiety and problems with behavioral control [12–14], which could be the consequences of inadequate PA, excessive screen-based media exposure, social isolation, and feelings of loneliness [15]. It is important for this vulnerable population to participate in a suitable and adapted type of PA to improve independent functioning, quality of life, and well-being [7]. Therefore, identifying and evaluating effective, evidence-based, and enjoyable exercise strategies aimed at improving health and fitness would have important clinical implications for the SEN population.

Among an array of exercise strategies, high-intensity interval training (HIIT) has emerged as a novel and time-efficient strategy for improving health-related fitness in children and adolescents compared with traditional training methods [16, 17]. It has attracted widespread attention among pediatric health and fitness professionals over the past decade [16, 17] and has been ranked among the top 10 in the American College of Sports Medicine Worldwide Survey of Fitness Trends since 2013 [18]. HIIT typically involves repeated short bouts of high-intensity exercise interspersed with active or inactive periods of recovery [19]. Its intermittent nature is more likely to be relevant to the sporadic, high-intensity, habitual activity patterns during childhood and adolescence than continuous, light-, and moderate-intensity exercise [17].

Recent systematic reviews exploring the efficacy of HIIT in promoting positive health-related outcomes in typically developing children and adolescents have been conducted [16, 17, 20, 21]. Overall, there is extensive evidence suggesting that HIIT is effective in improving physical fitness and cardiometabolic health [16, 17, 20] as well as mental health and cognitive performance in children and adolescents [21]. There is clear potential for the adaptation of evidence-based HIIT strategies (known to be effective in typically developing children and adolescents) for those with SEN, given the low levels of PA and fitness typically observed in the SEN cohort [5–8]. Some of the distinctive features of HIIT (e.g., time efficiency, inexpensive equipment, minimal space requirement, and variety of exercise selections) [22] may also facilitate participation in PA for children and adolescents with SEN [11]. Moreover, children and adolescents may less likely perform structured PA/ exercise training for the sake of it, but HIIT can be viable and sustainably incorporated as part of a sport (e.g., soccer, tennis, and athletics) or play in which participants enjoy [23]. However, it remains unclear whether the fitness and health outcomes following HIIT would be different in children and adolescents with or without SEN. Multiple factors, including biological, environmental, and social factors, surrounding the SEN cohort could have significant impacts on their PA behaviors [8] and, hence, subsequent HIIT outcomes. Furthermore, there is an understandable concern about the feasibility (e.g., safety and adherence) of HIIT in children and adolescents with SEN, which has yet to be thoroughly evaluated in the literature. These knowledge gaps should be filled before HIIT programs can be fully recommended for the SEN population. To the best of our knowledge, no systematic review has investigated the efficacy of HIIT in children and adolescents with different types of SEN. Therefore, this review aimed to systematically synthesize the scientific literature on HIIT in improving health-related fitness, mental health, and cognitive performance in children and adolescents with SEN.

## Methods

### Search strategy

This systematic review was performed in accordance with the PRISMA statement [24] and registered in the PROSPERO database (CRD42022352696). Electronic database searches were performed in MEDLINE, Embase, SPORTDiscus, Web of Science, Scopus, PsycINFO, CINAHL, and the Cochrane Library, using all available records up to August 10, 2022. The search terms covered the areas of HIIT, children and adolescents, and various types of SEN (e.g., attention deficit hyperactivity disorder [ADHD], cerebral palsy, developmental coordination disorder, and mental illness). We used the operation guide for integrated education by the HKSAR Government. The detailed search strategy is presented in Supplement 1.

### Selection procedure and eligibility criteria

After all duplicates were removed, two reviewers (EP and WW) independently screened the titles, abstracts, and full texts of the searched studies using predetermined criteria. Inclusion criteria for eligible studies were as follows: studies that 1) included a structured HIIT protocol (i.e., ≥ 80% maximum heart rate [HR_max_] or peak oxygen uptake) delivered in any setting (e.g., school, laboratory, or community facility); 2) quantitively measured and reported at least one outcome of physical fitness-related parameters (e.g., body composition, cardiorespiratory fitness, muscular fitness, anaerobic performance, functional capacity, and motor proficiency), cardiometabolic risk biomarkers (e.g., blood pressure, lipid profile, and glycemic responses), mental health (e.g., self-perception, mental wellness, ill-being, and mood states), and cognitive performance (e.g., executive function); 3) examined children or adolescents aged 5–17 years with SEN; 4) were randomized and non-randomized experimental studies (both chronic and acute studies); and 5) were published in a peer-reviewed journal with full text in English. The exclusion criteria were as follows: 1) studies involving adult participants, and 2) cross-sectional or longitudinal studies that did not evaluate an HIIT protocol.

Inter-reviewer disagreements were resolved by consensus or arbitration by a third reviewer (FS). Eligible studies were collected and imported into EndNote X10. Where the full manuscript was not available, the corresponding author was contacted via mail. The reference lists of the selected manuscripts were examined for other potentially eligible papers.

### Assessment of risk of bias

The revised Cochrane risk-of-bias tool for randomized trials (RoB 2) [25] and the Risk of Bias in Non-randomized Studies of Interventions (ROBINS-I) [26] were used to gauge the risk of bias in the findings of the included randomized and non-randomized studies, respectively. RoB 2 addresses five bias domains: randomization, deviations from intended interventions, missing outcome data, and measurement and selection of reported results. Each domain was judged as “low risk,” “some concerns,” or “high risk” based on responses to signaling questions, resulting in an overall bias judgement for the specific study outcome being assessed. Similarly, the ROBINS-I tool covers seven domains, including confounding, selection, measurement of intervention, missing data, selection of reported results, measurement of outcomes, and reported results, through which bias may be introduced in a non-randomized study. The judgement within each domain was categorized as low, moderate, serious, or critical risk of bias based on responses to signaling questions, leading to an overall risk of bias judgement for the outcome being assessed. Two authors (EP and WW) independently determined the risk of bias, and all disagreements were resolved by a third researcher (FS).

### Data extraction

A data extraction table has been developed (Table 1). The extracted data included the lead author, year of publication, study location (country of origin), population characteristics (children or adolescents and type of SEN), intervention protocols, frequency, and duration. One reviewer (EP) extracted the aforementioned information, which was then verified by a second reviewer (WW).

**Table 1:** Summary of characteristics of all studies meeting the inclusion criteria

Study	Population	Age (year)	Group	Size (n)	Protocol	Setting	Duration	Frequency (d/wk)
Boer et al*.* 2014 [27] Belgium RCT	Adolescents with intellectual disabilities; *N* = 54 (30 boys)	17.0 ± 3.0	HIIT	17	Week 1–7: 10 × 15 s sprint bouts at a resistance matching with the ventilatory threshold interspersed with 45 s rest. Week 8–15: 10 × 15 s sprint bouts at 110% ventilatory threshold interspersed with 45 s rest	Supervised by physiotherapists at schools	15 weeks	2
			CAT	15	three blocks of 10 min continuous training			
			CON	14	no supervised exercise training			
Braaksma et al. 2022 [28] Netherlands Non-RCT	Children with developmental coordination disorder; *N* = 20 (16 boys)	10.0 ± 1.6	HIIT	20	Based on running, strength exercises and plyometrics, ≥ 80% HR_max_	Supervised by trained physical therapists and PE teachers at rehabilitation centres or special schools	10 weeks	2
Lauglo et al*.* 2016 [29] Norway Non-RCT	Children with cerebral palsy; *N* = 20 (11 boys)	13–16	HIIT	14	4 × 4 min intervals at 85% HR_max_ interspersed with active recovery at about 70% of HR_max_ on a treadmill	Supervised by physiotherapists Venue not reported	5–12 weeks	2–4
Leahy et al*.* 2021 [30] Australia non-RCT	Adolescents with disability; *N* = 11 (7 boys)	17.3 ± 0.7	HIIT	16	~ 10 min and involves 8 × 30 s low complexity exercise interspersed with 30 s rest, ≥ 85% age-predicted HR_max_	Supervised by teachers at schools	2 months	2–3
Lee et al*.* 2019 [31] Canada Randomized crossover	Adolescents hospitalized for a mental illness; *N* = 28 (8 boys)	15.5 ± 0.9	HIIT	28	12 min HIIT circuit consisting of body weight exercises performed in a 1:1 work to rest ratio	Screened by psychiatrists and nurses at a hospital	1 day (acute effect)	N.A.
			CON	28	reading magazines			
Messler et al*.* 2018 [32] Germany RCT	Boys with ADHD; *N* = *18*	11.0 ± 1.0	HIIT	14	4 × 4 min intervals at 95% HR_max_ interspersed with 3 min recovery at < 60% HR_max_	Recommended by physician/psychologist at a hospital	3 weeks	3
			TRAD	14	60 min sessions of ball and team games, court sports, and climbing at < 70% HR_max_)			
Schranz et al*.* 2018 [33] Austria RCT	Children with cerebral palsy; *N* = 22 (15 boys)	13.4 ± 2.4 (HIIT) 12.2 ± 2.7 (PRT)	HIIT	11	3 rounds of 5 functional exercises with maximal intensity in short intervals of 30 s, interspersed with 30 s rest	Home-based workout with DVD instructions	8 weeks	3
			PRT	11	same functional exercises, intensity was progressively increased using a weight vest			
Smati et al*.* 2022 [34] Canada Non-RCT	Children with cerebral palsy with GMFCS level III–IV; *N* = 9 (5 boys)	8.7 ± 1.7	HIIT	9	physical activities/ circuit training exercises mainly involved short sprints or fast walking (10–15 s) interspersed walking recovery period at self-selected speed (30–60 s)	Supervised by PE teachers and undergraduate students in kinesiology at a school	12 weeks	3
Soori et al*.* 2020 [35] Iran RCT	Adolescents with ADHD; *N* = 43 (20 boys)	12.6 ± 0.2 (HIIT) 12.5 ± 0.5 (CON)	HIIT	16	20 m running program repetitions interspersed with 20–30 s rest, ≥ 85% HR_max_	Not reported	6 weeks	3
			CON	17	maintained their daily activities			
Taylor et al*.* 2019 [36] Australia Non-RCT	Adolescents with serious mental illness; *N* = 30 (11 boys)	16.0 ± 1.2	HIIT	15	4 × 30 s maximal cycling sprints interspersed with 4 min recovery	Supervised by researchers at a hospital	8 weeks	3
			CON	15	received treatment as usual			
Torabi et al*.* 2018 [37] Iran Non-RCT	Adolescents with ADHD; *N* = 50 (30 boys)	12.7 ± 1.1	HIIT	25	20 m running program repetitions interspersed with 20–30 s rest, ≥ 85% HR_max_	Supervised by researchers at laboratories	6 weeks	3
			CON	25	no training throughout the experimental period			
Wymbs et al.2021 [38] USA Crossover	Children with ADHD; *N* = 78 (57 boys)	9.7 ± 2.5	HIIT	78	~ 25 min in total, consisted of short bursts (2–5 min) of aerobic and anaerobic activity (e.g. running and doing jumping jacks) at 80–90% HR_max_, interspersed with 2 min recovery	Supervised by undergraduate and graduate students trained by psychologists at a therapeutic summer camp	15 days	7
			CON	78	~ 25 min in total, consisted of short bouts (2–5 min) of low intensity activities (e.g. walking, yoga) at 50–75% HR_max_, interspersed with 2 min recovery (self-controlled, children participated in high or low intensity exercise on alternating days)			
Zwinkels et al. 2018 [39] Netherlands Non-RCT	Youth with physical disabilities *N* = 70 (38 boys)	13.4 ± 2.9	HIIT-runners	36	30 s all-out exercises interspersed with 90–120 s active recovery	Supervised by physical educators and/or physical therapists at schools	8 weeks	2
			HIIT-walkers	25	30 s all-out exercises interspersed with 90–120 s active recovery			
			HIIT- wheelchair users	9	30 s all-out exercises interspersed with 90–120 s active recovery			

*CAT* continuous aerobic training*, CON* control group*, HR*_*max*_ maximum heart rate, *PRT* progressive resistance training, *RCT* randomized controlled trial

Cohen’s *d* was used to determine the standardized effect sizes (ES) of HIIT interventions on the reported outcome measures, where appropriate [40]. ES of 0.2, 0.5, and > 0.8 were regarded as small, moderate, and large effect sizes, respectively; a score of < 0.2 was considered to be negligible.

### Heterogeneity assessment

Because of variations in the characteristics of the studies included in this review, for example, among interventions, outcome measures, and cohort populations (i.e., various types of SEN), amalgamating the results of a meta-analysis was deemed unsuitable. Therefore, the results of this review were analyzed narratively.

## Results

### Study selection

**Fig. 1:**
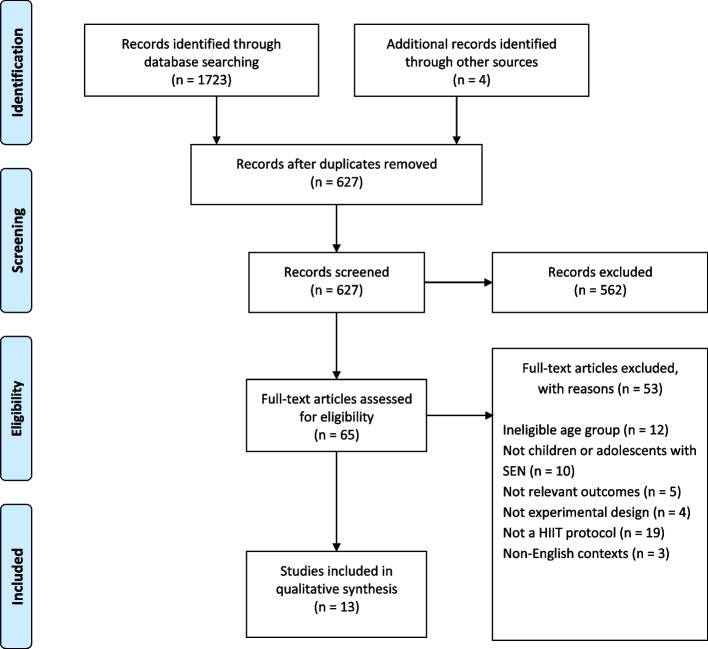
Flow diagram of outcomes of review (study flowchart)

The search strategy identified 1723 articles from eight electronic databases, and 4 other articles were manually identified. After removing duplicates, 627 articles remained, 562 of which were subsequently excluded after their titles and abstracts were screened. Of the 65 remaining full-text articles, 13 fulfilled the inclusion criteria (Fig. 1).

### Characteristics of included studies

A summary of the author, year, country, participant characteristics, and study design is presented in Table 1. The sample sizes of the 13 studies ranged from 9 [34] to 78 [38], and 453 participants were included in the overall review. The age of participants ranged from 5 to 18 years, with the youngest mean age being 8.7 ± 1.7 years [34] and the oldest being 17.3 ± 0.7 years [30]. There were four randomized controlled trials [27, 32, 33, 35], one randomized crossover trial [31], and eight quasi-experimental trials [28–30, 34, 36–39]. The included studies were conducted mainly in Western countries. Two studies each were conducted in the Netherlands [28, 39], Australia [30, 36], Canada [31, 34] and Iran [35, 37] and one each in Belgium [27], Germany [32], Norway [29], Austria [33] and the United States [38]. Furthermore, six types of SEN were included in this review: four papers discussed ADHD [32, 35, 37, 38], three discussed cerebral palsy [29, 33], two discussed mental illness [31, 36], one discussed physical disability [39], one discussed intellectual disability [27], and one discussed developmental coordination disorder [28]. Additionally, one study focused on several types of disabilities [30].

The exercise protocols are summarized in Table 1. Studies have used various PA modalities to engage children and adolescents in HIIT, including short bouts of sprinting [27, 34, 36, 39], functional exercise [33], running [29, 35, 37], circuit training [31], low-complexity exercise [30], and a combination of aerobic and anaerobic activities (e.g., running and jumping jacks) [38]. One study included a combination of modalities (e.g., running, strength exercises, and plyometrics) [28], whereas another did not specify the modality of the HIIT protocol [32]. Five studies were conducted in school settings [27, 28, 30, 34, 39], three in hospitals [31, 32, 36], and one each in laboratories [37], home environment [33] and a therapeutic summer camp [38]. All interventions ranged in duration from 2 to 15 weeks, with the exception of one acute study [31]. The interventions generally had a frequency of 2–4 days per week.

### Risk of bias

The methodological rigor of the studies included in this review according to the risk of bias assessment is presented in Table 2 (RoB 2) and Table 3 (ROBINS-I). Among the five randomized studies included in RoB 2, three displayed a “high” risk of overall bias [31, 33, 35], mainly because of a significant portion of missing outcome data (i.e., high dropout rates). Two studies displayed “some concerns” regarding overall bias [27, 32] arising from potential deviations from intended interventions and selective reporting of results. Of the eight included non-randomized studies in the ROBINS-I, six demonstrated a “serious” overall risk of bias, mainly due to baseline confounding, measurement of outcomes, and selection of the reported result [28–30, 34, 36, 39]. One study showed a “moderate” risk of overall bias arising from a lack of adherence data [37]. Another study displayed a “low” risk of overall bias, showing low risks in most bias domains [38].

**Table 2:** Risk of bias assessment using cochrane risk-of-bias tool for randomized trials (RoB 2)

Study	Randomization process	Deviations from intended interventions	Missing outcome data	Measurement of the outcome	Selection of the reported result	Overall bias
Boer et al. 2014 [27]	Low	Some concerns	Low	Low	Some concerns	Some concerns
Lee et al. 2019 [31]	Low	Low	High	Low	Some concerns	High
Schranz et al. 2018 [33]	Low	High	High	Low	Some concerns	High
Soori et al. 2020 [35]	Low	High	High	Low	Some concerns	High
Messler et al. 2018 [32]	Some concerns	Some concerns	Low	Low	Some concerns	Some concerns

**Table 3:** Risk of bias assessment using The Risk Of Bias In Non-randomized Studies of Interventions (ROBINS-I) assessment tool

Study	Confounding	Selection	Measurement of intervention	Missing data	Selection of the reported result	Measurement of outcomes	Reported result	Overall
Braaksma et al. 2022 [28]	Serious	Low	Low	Moderate	Low	Serious	Serious	Serious
Lauglo et al. 2016 [29]	Serious	Low	Low	Moderate	Moderate	Serious	Serious	Serious
Leahy et al. 2021 [30]	Serious	Low	Low	Moderate	Moderate	Serious	Serious	Serious
Smati et al. 2022 [34]	Serious	Low	Low	Moderate	Low	Serious	Serious	Serious
Taylor et al. 2019 [36]	Serious	Moderate	Low	Moderate	Low	Moderate	Moderate	Serious
Torabi et al. 2018 [37]	Moderate	Low	Low	Moderate	Low	Moderate	Moderate	Moderate
Wymbs et al. 2021 [38]	Low	Low	Low	Low	Low	Moderate	Moderate	Low
Zwinkels et al. 2018 [39]	Serious	Low	Low	Moderate	Serious	Serious	Serious	Serious

### Outcome measures

A summary of the results of all 13 studies is presented in Supplement 2. Six studies reported the effect of HIIT on body composition parameters, including body mass index (BMI) (*n* = 6; ES =  − 0.55 to 0.02), waist circumference (*n* = 3; ES =  − 0.33 to 0.01), body fat percentage (*n* = 3; ES =  − 0.55 to − 0.14), and fat mass (*n* = 4; ES =  − 0.51 to 0.00), with the majority concluding that HIIT had significant benefits (Table 4). Twelve of the included studies reported the effect of HIIT on physical fitness-related outcomes, including cardiorespiratory fitness (*n* = 6; ES =  − 0.02 to 0.68), muscular fitness (*n* = 5; ES =  − 0.22 to 0.91), anaerobic performance (*n* = 4; ES =  − 0.04 to 0.42), functional capacity (*n* = 4; ES = 0.09 to 1.42), and motor proficiency (*n* = 1; ES = 0.73) (Table 5). Improvements in various fitness tests, such as peak oxygen uptake assessment (i.e., shuttle run test and incremental test using cycle ergometers and treadmills), muscle power sprint test, and 6-min walk test, were consistently observed following HIIT interventions. However, improvements in muscular fitness tests, such as the handgrip strength test, were less conclusive.

**Table 4:** Baseline to post-intervention changes in common measures of body composition

Study	Outcome measure	HIIT Baseline Mean ± SD	HIIT Post-test Mean ± SD	Mean change (from baseline)	Effect size (Cohen’s *d*)
Boer et al. 2014 [27]	BMI	28.4 ± 4.7	27.7 ± 4.7	–0.7	–0.15
Lauglo et al. 2016 [29]	BMI	21.0	21.4	0.4	N.A.
Soori et al. 2022 [35]	BMI (z-score)	1.63 ± 0.27	1.43 ± 0.66	–0.2	–0.40
Taylor et al. 2019 [36]	BMI	26.0 ± 7.0	25.7 ± 7.1	–0.3	–0.04
Torabi et al. 2018 [37]	BMI (boys only)	24.4 ± 3.5	23.6 ± 3.8	–0.8	–0.22
Torabi et al. 2018 [37]	BMI (girls only)	26.7 ± 2.6	25.4 ± 2.1	–1.3	–0.55
Zwinkels et al. 2019 [39]	BMI	22.2 ± 4.8	22.3 ± 5.1	0.1	0.02
Zwinkels et al. 2019 [39]	BMI (z-score)	1.31 ± 1.4	1.29 ± 1.4	–0.02	–0.01
Boer et al. 2014 [27]	Waist circumference (cm)	95.8 ± 13.1	91.5 ± 13.1	–4.3	–0.33
Taylor et al. 2019 [36]	Waist circumference (cm)	81.0 ± 13.7	80.3 ± 13.6	–0.7	–0.05
Zwinkels et al. 2019 [39]	Waist circumference (cm)	79.2 ± 14.5	79.4 ± 14.1	0.2	0.01
Boer et al. 2014 [27]	Body fat (%)	34.2 ± 6.9	30.4 ± 7.0	–3.8	–0.55
Lauglo et al. 2016 [29]	Body fat (%)	30.9	29.9	1.0	N.A.
Taylor et al. 2019 [36]	Body fat (%)	30.2 ± 10.6	28.7 ± 11.2	–1.5	–0.14
Lauglo et al. 2016 [29]	Fat mass (kg)	15.7	16.2	0.5	N.A.
Soori et al. 2022 [35]	Fat mass (kg)	27.9 ± 5.9	26.0 ± 5.7	–1.9	–0.32
Torabi et al. 2018 [37]	Fat mass (kg, boys only)	25.5 ± 5.9	22.6 ± 5.5	–2.9	–0.51
Torabi et al. 2018 [37]	Fat mass (kg, girls only)	29.1 ± 5.8	27.8 ± 5	–1.3	–0.24
Zwinkels et al. 2019 [39]	Fat mass (kg)	30.4 ± 10.4	30.4 ± 10.4	0	0

*BMI* body mass index

**Table 5:** Baseline to post-intervention changes in common measures of physical fitness related outcomes

Study	Outcome measure	HIIT Baseline Mean ± SD	HIIT Post-test Mean ± SD	Mean change (from baseline)	Effect size (Cohen’s *d*)
Boer et al. 2014 [27]	VO_2peak_ (mL/kg/min)	31.5 ± 5.2	31.4 ± 4.8	–0.1	–0.02
Braaksma et al. 2022 [28]	VO_2peak_ (mL/kg/min)	42.3 ± 4.3	43.7 ± 4.3	1.4	0.32
Lauglo et al. 2016 [29]	VO_2peak_ (mL/kg/min)	37.3	41.0	3.7	N.A.
Messler et al. 2018 [32]	VO_2peak_ (L/min)	1.25 ± 0.37	1.31 ± 0.34	0.06	0.17
Taylor et al. 2019 [36]	VO_2peak_ (mL/kg/min)	23.3 ± 6.1	29.1 ± 10.4	5.8	0.68
Zwinkels et al. 2019 [39]	VO_2peak_ (mL/kg/min)	37.6 ± 9.7	37.7 ± 8.8	0.1	0.01
Boer et al. 2014 [27]	6MWT (m)	598 ± 64.0	666 ± 69.4	67.7	1.02
Leahy et al. 2021 [30]	6MWT (m)	400 ± 127	563 ± 158	163	1.25
Schranz et al. 2018 [33]	6MWT (m)	568 ± 65	573 ± 58	5	0.09
Smati et al. 2022 [34]	6MWT (m)	199 ± 48.6	317 ± 107	118	1.41
Boer et al. 2014 [27]	Sit-to-stand (repetitions)	16.8 ± 4.0	16.0 ± 3.4	–0.8	–0.22
Leahy et al. 2021 [30]	Sit-to-stand (repetitions)	15 ± 5	18 ± 5	3	0.91
Leahy et al. 2021 [30]	Push-up (repetitions)	5 ± 6	12 ± 12	7	0.99
Braaksma et al. 2022 [28]	Grip strength (kg)	14.5 ± 5.4	14.9 ± 5.5	0.2	0.04
Zwinkels et al. 2019 [39]	Grip strength (N)	151 ± 76.3	150 ± 72.3	–1	–0.01
Zwinkels et al. 2019 [39]	Standing broad jump (m)	87.4 ± 35.6	91.7 ± 38.7	4.3	0.33
Braaksma et al. 2022 [28]	Anaerobic performance (MPST mean power, W)	163 ± 73	198 ± 89	35	0.42
Taylor et al. 2019 [36]	Anaerobic performance (Wingate peak power, W)	342 ± 145	392 ± 152	50	0.34
Taylor et al. 2019 [36]	Anaerobic performance (Wingate mean power, W)	234 ± 99	230 ± 93	–4	–0.04
Zwinkels et al. 2019 [39]	Anaerobic performance (MPST peak power, W)	199 ± 161	222 ± 188	23	0.33
Zwinkels et al. 2019 [39]	Anaerobic performance (MPST mean power, W)	169 ± 136	187 ± 156	18	0.40

*6MWT* 6-min walk test, *MPST* muscle power sprint test, *VO*_*2peak*_ Peak oxygen uptake

Biomarkers of cardiometabolic risk, including blood pressure (*n* = 3; ES =  − 1.22 to 0.45), lipid profile (*n* = 2; ES =  − 0.88 to 0.36), fasting blood glucose levels (*n* = 2; ES =  − 0.14 to 0.16), insulin (*n* = 1; ES =  − 0.60), and insulin resistance (*n* = 2; ES =  − 1.46 to − 0.56), were measured in five studies (Table 6). While significant improvements in insulin resistance and lipid profile (i.e., cholesterol and triglyceride levels) were consistently observed, findings on blood pressure tended to be inconclusive.

**Table 6:** Baseline to post-intervention changes in common measures of cardiometabolic risk biomarkers

Study	Outcome measure	Baseline Mean ± SD	Post-test Mean ± SD	Mean change (from baseline)	Effect size (Cohen’s *d*)
Boer et al. 2014 [27]	SBP (mmHg)	124 ± 10	113 ± 8	–11	–1.22
Taylor et al. 2019 [36]	SBP (mmHg)	107 ± 6.60	111 ± 10.36	3.91	0.45
Zwinkels et al. 2019 [39]	SBP (mmHg)	123 ± 14.0	120 ± 12.8	–3	–0.34
Boer et al. 2014 [27]	DBP (mmHg)	74 ± 7	77 ± 8	3	0.40
Taylor et al. 2019 [36]	DBP (mmHg)	68.2 ± 5.39	70.4 ± 6.05	2.2	0.39
Zwinkels et al. 2019 [39]	DBP (mmHg)	67.8 ± 10.3	65.4 ± 8.5	–2.4	–0.29
Boer et al. 2014 [27]	Total cholesterol (mg/dL)	170 ± 25	155 ± 23	–15	–0.62
Zwinkels et al. 2019 [39]	Total cholesterol (mmol/L)	3.8 ± 0.67	3.81 ± 0.68	0.01	0.01
Boer et al. 2014 [27]	HDL-cholesterol (mg/dL)	54.9 ± 13.5	59.4 ± 11.4	4.5	0.36
Zwinkels et al. 2019 [39]	HDL-cholesterol (mmol/L)	1.23 ± 0.36	1.25 ± 0.36	0.02	0.06
Boer et al. 2014 [27]	LDL-cholesterol (mg/dL)	105 ± 12.0	96 ± 9.3	–9	–0.88
Zwinkels et al. 2019 [39]	LDL-cholesterol (mmol/L)	2.25 ± 0.56	2.2 ± 0.51	–0.05	–0.09
Boer et al. 2014 [27]	Triglycerides (mg/dL)	79.2 ± 22.2	70.8 ± 16.7	–8.4	–0.43
Zwinkels et al. 2019 [39]	Triglyceride (mmol/L)	1.01 ± 0.59	1.11 ± 0.68	0.1	0.16
Boer et al. 2014 [27]	Glucose (mg/dL)	86 ± 7.6	85 ± 7.1	–1	–0.14
Zwinkels et al. 2019 [39]	Glucose (mmol/L)	4.68 ± 0.61	4.8 ± 0.58	0.1	0.16
Boer et al. 2014 [27]	Insulin (IU/mg)	14 ± 5.9	11 ± 4.0	–3	–0.60
Torabi et al. 2018 [37]	Insulin resistance (HOMA-IR, boys only)	3.6 ± 0.9	2.6 ± 0.6	–1	1.31
Torabi et al. 2018 [37]	Insulin resistance (HOMA-IR, girls only)	3.3 ± 0.8	2.2 ± 0.7	–1.1	–1.46
Boer et al. 2014 [27]	Insulin resistance (HOMA-IR)	2.9 ± 1.3	2.3 ± 0.8	–0.6	–0.56

*DBP* diastolic blood pressure, *HDL* high-density lipoprotein, *HOMA-IR* Homeostatic model assessment of insulin resistance, *LDL* low-density lipoprotein, *SBP* systolic blood pressure

Nine of the included studies examined mental health- or cognitive performance-related outcomes, including mood (*n* = 2), quality of life (*n* = 3), well-being index (*n* = 1), social behavior (*n* = 4), and inhibitory control (*n* = 1). Eight of the nine studies showed improvements in these outcomes using various subjective and objective measures (e.g., questionnaires, rating scales, cognitive tests, and parents’ observations). The only exception was the study by Wymbs et al. [38], in which children with ADHD had a wider range of behavioral problems immediately after HIIT and showed worse initial mood and more negative mood changes over time (Supplement 2).

### Adherence and adverse events

Four studies reported no adverse events using HIIT protocols throughout the experimental period [27, 29, 30, 39]. Six studies reported on adherence to HIIT protocols [28, 33–36, 39] and the overall adherence level was satisfactory (i.e., 70–100%). However, several studies did not report either intervention adherence or adverse events [31, 32, 35, 37, 38].

## Discussion

The present review aimed to synthesize available evidence regarding the efficacy of HIIT in children and adolescents with SEN. In general, our findings were consistent with those of previous HIIT studies in typically developing children and adolescents, showing benefits in physical fitness-related outcomes, as well as mental health and cognitive performance. This suggests that the benefits of HIIT are likely to be universal for all children and adolescents, including those with SEN.

### Effects of HIIT on body composition

Recent systematic reviews exploring the efficacy of HIIT in promoting favorable changes in body composition have shown promising results in children and adolescents [16, 17], including those who are overweight or obese [41]. The present findings revealed that children and adolescents with SEN also showed favorable changes in various body composition measures, including BMI, waist circumference, body fat percentage, and fat mass following HIIT interventions. These findings have significant clinical implications, as children and adolescents with SEN or disabilities are at a higher risk (up to 3–6 times) of being overweight and obese than their typically developing peers [9]. It has been proposed that HIIT induces direct energy consumption during exercise and that additional fat loss mechanisms might be involved owing to the intense nature of HIIT. These fat loss mechanisms include increased excess post-exercise oxygen consumption, decreased post-exercise appetite, and increased catecholamine release, which elevates tissue lipolysis [42]. An additional benefit of HIIT is that the same fat loss effects can be obtained with a significantly shorter exercise duration.

### Effects of HIIT on physical fitness-related outcomes

Our results revealed that HIIT generally elicited positive changes in a range of physical fitness-related outcomes, including cardiorespiratory fitness, anaerobic performance, functional capacity, and motor proficiency among the SEN cohort. These findings are of paramount importance given that children and adolescents with SEN are more likely to face different physical barriers in their daily lives, which may affect their independent functioning and increase the burden on caregivers [11, 43]. Our results demonstrate that following HIIT, positive outcomes on performance-related fitness, such as improved exercise capacity, sprint performance, and agility, can be expected. Such performance-related fitness enhancements are also related to functional performance in daily life. In particular, the intermittent nature of HIIT (i.e., a mixture of low- and high-intensity exercises) with frequent explosive movements requires substantial neuromuscular loads and contributions from both the anaerobic and aerobic pathways. This is more likely to be relevant to activity patterns during childhood and adolescence than continuous-based exercise [17]. Regarding muscular fitness, it is interesting to note that improvements in handgrip strength and sit-to-stand tests were not consistently observed in two of the included studies. Such findings may reflect a lack of training specificity in HIIT protocols that predominantly involve running and sprinting, which are likely to improve other fitness components (e.g., speed, cardiorespiratory fitness, and body composition) [16]. Future HIIT studies may consider using a more diverse HIIT protocol that targets major muscle groups in different parts of the body.

### Effects of HIIT on cardiometabolic risk biomarkers

In addition to its beneficial effects on physical fitness, our results also suggest that HIIT favors certain cardiometabolic risk biomarkers, as it consistently improves lipid profiles (i.e., cholesterol and triglyceride levels) and insulin resistance across multiple studies. Cardiometabolic health is an important issue, particularly among children and adolescents with SEN, as this cohort has been found to be insufficiently active and tends to adopt a physically inactive lifestyle [5–8]. This may put them at a higher risk for chronic cardiometabolic diseases, such as cerebrovascular disease, coronary artery disease, and type-2 diabetes, in adulthood [1]. The metabolic benefits of habitual HIIT are thought to be related to repeated acute responses to a single high-intensity exercise session [44]. For example, cardiometabolic changes typically observed after a bout of exercise are transient but can be experienced on a routine basis after regular exercise [44]. The proposed physiological mechanisms underlying the improvement in cardiometabolic health following HIIT have recently been outlined in detail elsewhere [13]. These include HIIT-induced improvements in glycemic responses through enhanced muscle oxidative capacity and increased skeletal muscle glucose transporter protein content [45, 46], which promote the overall glucose transport capacity of the body. Interestingly, our findings regarding changes in blood pressure and fasting glucose levels tend to be inconclusive. This may be because the baseline blood pressure and fasting glucose levels of participants in those related studies were within the normal range; hence, further improvements were less likely to be observed.

### Effects of HIIT on mental health and cognitive performance

It is well established that both acute and chronic PA can result in several physiological and psychological changes that elicit improvements in brain-based processes. Our results are in line with those of a recent review suggesting that HIIT can improve cognitive performance and mental health in children and adolescents [21]. Improvements in mood, quality of life, well-being index, and social, behavioral, and inhibitory control were reported in the included studies. For instance, Messler et al. [32] reported that 3 weeks of HIIT (4 × 4 min intervals at 95% HR_max_) was more effective than standard multimodal therapy in improving health-related quality of life, competence, and behavioral symptoms (i.e., attention) in boys with ADHD. Taylor et al. [36] demonstrated that an 8-week bicycle-based HIIT intervention (4 × 30 s maximal sprints) helped protect and potentially improve multiple health indices (e.g., psychiatric symptoms and mental well-being) in adolescents with serious mental illness. Using a randomized, counterbalanced study design, Lee et al. [31] reported that an acute bout of a 12-min HIIT circuit improved inhibitory control by increasing response efficiency in adolescents hospitalized for mental illness. A psychophysiological mechanism has been identified to underlie this positive relationship, suggesting that higher concentrations of several neurochemicals (i.e., brain-derived neurotrophic factor and catecholamines [e.g., dopamine, epinephrine]) induced by exercise, particularly high-intensity exercise (i.e., HIIT), may improve cognitive performance and psychological well-being, leading to better overall mental health and cognitive performance [47]. To date, most HIIT studies related to mental health and cognitive performance have been conducted in typically developing children and adolescents [21]; however, some studies have shown positive effects of PA on self-competence, quality of life, mental wellness, and enjoyment in children with SEN [48–51]. While the aforementioned studies did not exclusively focus on the effects of HIIT (but rather on any form of moderate-to-vigorous exercise), their results are in line with our findings, suggesting that HIIT may provide a time-efficient alternative to induce mental health and cognitive performance benefits.

### Potential moderators of HIIT outcomes

Owing to the relatively small sample size and methodological limitations of the studies included in our review, we were unable to perform quantitative subgroup analyses for potential moderators. However, this could have implications for future research, given the possibility that the effects of HIIT on fitness and health outcomes may depend on biological factors, such as age, sex, and type of disabilities in the SEN cohort [8]. Our data show that the beneficial effects associated with HIIT appear to be consistent across different sexes, pubertal stages, and settings for various types of SEN (e.g., ADHD, physical disability, and mental illness). Future studies should also explore how various intervention components (e.g., type, intensity, duration, and frequency of exercise) may moderate HIIT outcomes to elucidate the most effective protocol.

### Safety precautions and adherence

There is an understandable concern about safety and adherence to HIIT in children and adolescents with SEN. Although four of our included studies reported no adverse events and six reported relatively high adherence levels to HIIT interventions, several other studies did not report intervention adherence and adverse events. Nonetheless, HIIT performed at very high intensities is reportedly safe, well-tolerated, and attainable, even when applied to clinical populations with low initial fitness [52–54]. The safety concerns associated with HIIT among children and adolescents do not seem to be significantly greater than those associated with traditional programs [55]. Furthermore, the built-in recovery periods of HIIT may be relevant to the sporadic and highly intense nature of children’s habitual play patterns. This may ease feelings of displeasure during workout sessions by reducing boredom and inducing a sense of accomplishment after each interval, thus enhancing participants’ motivation in the long run [56]. That said, children and adolescents with SEN should be encouraged to undergo medical evaluations prior to the initiation of any exercise program [2, 3]. Fitness and health professionals should tailor the HIIT program to meet the needs and interest of children and adolescents with SEN. An example could be implementing HIIT as part of a sport or play during school PE lessons or leisure time. Programs should always be delivered in a progressive manner with adequate supervision to ensure long-term safety and adherence.

## Strengths and limitations

To the best of our knowledge, this is the first systematic review to examine the effects of HIIT on health-related outcomes and cognitive performance in children and adolescents with SEN. The strengths of our review include adherence to PRISMA guidelines and the use of widely recognized benchmarks (e.g., Cochrane RoB 2 and ROBINS-I tools) to assess the scientific rigor of the included studies. However, this review had several limitations. First, the included studies were relatively heterogeneous regarding SEN types and diversity in HIIT protocols (e.g., modality, work intensity, duration, volume, and setting). This hindered the extent to which the studies could be integrated and interpreted (e.g., performing separate quantitative analyses across subgroups), thereby limiting the generalizability of our findings. Second, only English-language articles were considered in the present review; hence, some relevant studies in other languages might have been overlooked. Moreover, a relatively high proportion of studies displayed a “high” or “serious” overall risk of bias, owing to a significant portion of missing outcome data and potential deviation from intended interventions. Future high-quality randomized controlled studies are warranted, and researchers should adopt an appropriate level of supervision to minimize dropout rates and control for confounding factors (e.g., participants’ daily PA and diet during the intervention). However, we believe that our findings provide valuable insights into the real-world application of HIIT in children and adolescents with SEN. From a practical perspective, our results suggest that HIIT can serve as a viable exercise option for enhancing fitness, health-related outcomes, and mental and cognitive performance in the SEN cohort. Further research investigating the benefits of HIIT can be conducted in a wider range of SEN populations (e.g., autism spectrum disorder). This will help provide more information on the safety and efficacy of different HIIT modes in each specific SEN cohort. Future studies should incorporate a follow-up period within the study design to evaluate the long-term sustainability of the HIIT-elicited benefits.

## Conclusion

In summary, the present review revealed that HIIT generally improved physical fitness, cardiometabolic risk biomarkers, mental health, and cognitive performance across a spectrum of SEN (e.g., ADHD, cerebral palsy, developmental coordination disorder, and mental illness). This study provides up-to-date evidence for HIIT as a viable exercise strategy for children and adolescents with SEN.

## Supplementary Information

**Additional file 1:** **Supplement 1. **Search strategy.

**Additional file 2:** **Supplement 2. **Summary of results of all included studies.

## Data Availability

Data sharing is not applicable to this article as no datasets were generated or analyzed during the current study.

## Abbreviations

ADHD: Attention deficit hyperactivity disorder
BMI: Body mass index
ES: Effect size
HIIT: High-intensity interval training
PA: Physical activity
PRISMA: Preferred Reporting Items for Systematic Reviews and Meta-Analyses
RoB 2: Revised Cochrane risk-of-bias tool for randomized trials
ROBINS: Risk of Bias in Non-randomized Studies of Interventions
SEN: Special educational needs

## References

[1] WHO. World Health Organization Physical Activity Fact Sheet. 2016.

[2] ACSM. ACSM's Guidelines for Exercise Testing and Prescription (11th Ed). Philadelphia, PA Wolters Kluwer; 2021.

[3] Bull FC, Al-Ansari SS, Biddle S, Borodulin K, Buman MP, Cardon G (2020). World Health Organization 2020 guidelines on physical activity and sedentary behaviour. Br J Sports Med.

[4] Ekelund U, Luan J, Sherar LB, Esliger DW, Griew P, Cooper A (2012). Moderate to vigorous physical activity and sedentary time and cardiometabolic risk factors in children and adolescents. JAMA.

[5] Sit CHP, Mckenzie TL, Cerin E, Chow BC, Huang WY, Yu J (2017). Physical activity and sedentary time among children with disabilities at school. Med Sci Sports Exerc.

[6] Corvey K, Menear KS, Preskitt J, Goldfarb S, Menachemi N (2016). Obesity, physical activity and sedentary behaviors in children with an autism spectrum disorder. Matern Child Health J.

[7] Rimmer JH, Marques AC (2012). Physical activity for people with disabilities. Lancet.

[8] Jung J, Leung W, Schram BM, Yun J (2018). Meta-analysis of physical activity levels in youth with and without disabilities. Adapt Phys Activ Q.

[9] Neter JE, Schokker DF, de Jong E, Renders CM, Seidell JC, Visscher TL (2011). The prevalence of overweight and obesity and its determinants in children with and without disabilities. J Pediatr.

[10] UNESCO. Revision of the International Standard Classification of Education (ISCED) 2011 [Available from: https://unterm.un.org/unterm/display/record/UNESCO/NA/5450bbef-11bd-437a-a2cd-df2cfa1d5852.

[11] Shields N, Synnot A (2016). Perceived barriers and facilitators to participation in physical activity for children with disability: a qualitative study. BMC Pediatr.

[12] Downs J, Blackmore AM, Epstein A, Skoss R, Langdon K, Jacoby P (2018). The prevalence of mental health disorders and symptoms in children and adolescents with cerebral palsy: a systematic review and meta-analysis. Dev Med Child Neurol.

[13] Emerson E, Einfeld S, Stancliffe RJ (2010). The mental health of young children with intellectual disabilities or borderline intellectual functioning. Soc Psych Psych Epid.

[14] Licence L, Oliver C, Moss J, Richards C (2020). Prevalence and risk-markers of self-harm in autistic children and adults. J Autism Dev Disord.

[15] Rodriguez-Ayllon M, Cadenas-Sanchez C, Estevez-Lopez F, Munoz NE, Mora-Gonzalez J, Migueles JH (2019). Role of physical activity and sedentary behavior in the mental health of preschoolers, children and adolescents: a systematic review and meta-analysis. Sports Med.

[16] Costigan SA, Eather N, Plotnikoff RC, Taaffe DR, Lubans DR (2015). High-intensity interval training for improving health-related fitness in adolescents: a systematic review and meta-analysis. Br J Sports Med.

[17] Eddolls WTB, McNarry MA, Stratton G, Winn CON, Mackintosh KA (2017). High-intensity interval training interventions in children and adolescents: a systematic review. Sports Med.

[18] Thompson W (2021). Worldwide survey of fitness trends for 2022. ACSM's Health Fit J.

[19] MacInnis MJ, Gibala MJ (2017). Physiological adaptations to interval training and the role of exercise intensity. J Physiol.

[20] Bauer N, Sperlich B, Holmberg HC, Engel FA (2022). Effects of high-intensity interval training in school on the physical performance and health of children and adolescents: a systematic review with meta-analysis. Sports Med Open.

[21] Leahy AA, Mavilidi MF, Smith JJ, Hillman CH, Eather N, Barker D (2020). Review of high-intensity interval training for cognitive and mental health in youth. Med Sci Sports Exerc.

[22] Klika B, Jordan C (2013). High-intensity circuit training using body weight: maximum results with minimal investment. ACSM's Health Fit J.

[23] Engel FA, Ackermann A, Chtourou H, Sperlich B (2018). High-intensity interval training performed by young athletes: a systematic review and meta-analysis. Front Physiol.

[24] Moher D, Liberati A, Tetzlaff J, Altman DG, Grp P (2009). Preferred reporting items for systematic reviews and meta-analyses: the PRISMA statement. J Clin Epidemiol.

[25] Minozzi S, Cinquini M, Gianola S, Gonzalez-Lorenzo M, Banzi R (2020). The revised Cochrane risk of bias tool for randomized trials (RoB 2) showed low interrater reliability and challenges in its application. J Clin Epidemiol.

[26] Sterne JA, Hernan MA, Reeves BC, Savovic J, Berkman ND, Viswanathan M (2016). ROBINS-I: a tool for assessing risk of bias in non-randomised studies of interventions. BMJ.

[27] Boer PH, Meeus M, Terblanche E, Rombaut L, De Wandele I, Hermans L (2014). The influence of sprint interval training on body composition, physical and metabolic fitness in adolescents and young adults with intellectual disability: a randomized controlled trial. Clin Rehabil.

[28] Braaksma P, Stuive I, van der Hoek FD, van der Sluis CK, Schoemaker MM, Dekker R (2018). We12BFit!-improving physical fitness in 7–12-year-old children with developmental coordination disorder: protocol of a multicenter single-arm mixed-method study. Front Pediatr.

[29] Lauglo R, Vik T, Lamvik T, Stensvold D, Finbraten AK, Moholdt T (2016). High-intensity interval training to improve fitness in children with cerebral palsy. BMJ Open Sport Exerc Med.

[30] Leahy AA, Kennedy SG, Smith JJ, Eather N, Boyer J, Thomas M (2021). Feasibility of a school-based physical activity intervention for adolescents with disability. Pilot Feasibility Stud.

[31] Lee JS, Boafo A, Greenham S, Longmuir PE. The effect of high-intensity interval training on inhibitory control in adolescents hospitalized for a mental illness. Ment Health Phys Act. 2019;17.

[32] Messler CF, Holmberg HC, Sperlich B (2018). Multimodal therapy involving high-intensity interval training improves the physical fitness, motor skills, social behavior, and quality of life of boys with ADHD: a randomized controlled study. J Atten Disord.

[33] Schranz C, Kruse A, Belohlavek T, Steinwender G, Tilp M, Pieber T (2018). Does home-based progressive resistance or high-intensity circuit training improve strength, function, activity or participation in children with cerebral palsy?. Arch Phys Med Rehabil.

[34] Smati S, Pouliot-Laforte A, Chevalier M, Lemay M, Ballaz L. Effect of power training on locomotion capacities in children with cerebral palsy with GMFCS level III-IV. Disabil Rehabil. 2022:1–7. 10.1080/09638288.2022.2090623 35737476

[35] Soori R, Goodarzvand F, Akbarnejad A, Effatpanah M, Ramezankhani A, Teixeira AL (2020). Effect of high-intensity interval training on clinical and laboratory parameters of adolescents with attention deficit hyperactivity disorder. Sci Sports.

[36] Taylor C, Sanders R, Hoon M, Starling J, Cobley S (2019). Can Sprint Interval Training (SIT) improve the psychological and physiological health of adolescents with SMI?. Evidence-Based Practice in Child and Adolescent Mental Health.

[37] Torabi F, Farahani A, Safakish S, Ramezankhani A, Dehghan F (2018). Evaluation of motor proficiency and adiponectin in adolescent students with attention deficit hyperactivity disorder after high-intensity intermittent training. Psychiatry Res.

[38] Wymbs FA, Wymbs B, Margherio S, Burd K (2021). The effects of high intensity versus low intensity exercise on academic productivity, mood, and behavior among youth with and without ADHD. J Child Fam Stud.

[39] Zwinkels M, Verschuren O, de Groot JF, Backx FJG, Wittink H, Visser-Meily A (2019). Effects of high-intensity interval training on fitness and health in youth with physical disabilities. Pediatr Phys Ther.

[40] Cohen J (1992). A Power Primer. Psychol Bull.

[41] Thivel D, Masurier J, Baquet G, Timmons BW, Pereira B, Berthoin S (2019). High-intensity interval training in overweight and obese children and adolescents: systematic review and meta-analysis. J Sports Med Phys Fitness.

[42] Irving BA, Davis CK, Brock DW, Weltman JY, Swift D, Barrett EJ (2008). Effect of exercise training intensity on abdominal visceral fat and body composition. Med Sci Sports Exerc.

[43] Bossink LWM, van der Putten AA, Vlaskamp C (2017). Understanding low levels of physical activity in people with intellectual disabilities: a systematic review to identify barriers and facilitators. Res Dev Disabil.

[44] Bond B, Weston KL, Williams CA, Barker AR (2017). Perspectives on high-intensity interval exercise for health promotion in children and adolescents. Open Access J Sports Med.

[45] Hood MS, Little JP, Tarnopolsky MA, Myslik F, Gibala MJ (2011). Low-volume interval training improves muscle oxidative capacity in sedentary adults. Med Sci Sports Exerc.

[46] Little JP, Gillen JB, Percival ME, Safdar A, Tarnopolsky MA, Punthakee Z (2011). Low-volume high-intensity interval training reduces hyperglycemia and increases muscle mitochondrial capacity in patients with type 2 diabetes. J Appl Physiol  (1985).

[47] Cooper SB, Dring KJ, Nevill ME (2016). High-Intensity intermittent exercise: effect on young people's cardiometabolic health and cognition. Curr Sports Med Rep.

[48] Yang W, Wong SHS, Sum RKW, Sit CHP (2021). The association between physical activity and mental health in children with special educational needs: a systematic review. Prev Med Rep.

[49] Martin JJ (2013). Benefits and barriers to physical activity for individuals with disabilities: a social-relational model of disability perspective. Disabil Rehabil.

[50] Sahlin KB, Lexell J (2015). Impact of organized sports on activity, participation, and quality of life in people with neurologic disabilities. Pm & R.

[51] Toscano CVA, Carvalho HM, Ferreira JP (2018). Exercise effects for children with autism spectrum disorder: metabolic health, autistic traits, and quality of life. Percept Mot Ski.

[52] Hussain SR, Macaluso A, Pearson SJ (2016). High-intensity interval training versus moderate-intensity continuous training in the prevention/management of cardiovascular disease. Cardiol Rev.

[53] Rognmo Ø, Moholdt T, Bakken H, Hole T, Mølstad P, Myhr NE (2012). Cardiovascular risk of high-versus moderate-intensity aerobic exercise in coronary heart disease patients. Circulation.

[54] Molnar AO, Eddeen AB, Ducharme R, Garg AX, Harel Z, McCallum MK (2017). Association of proteinuria and incident atrial fibrillation in patients with intact and reduced kidney function. J Am Heart Assoc.

[55] Lubans DR, Smith JJ, Eather N, Leahy AA, Morgan PJ, Lonsdale C (2020). Time-efficient intervention to improve older adolescents' cardiorespiratory fitness: findings from the 'Burn 2 Learn' cluster randomised controlled trial. Br J Sports Med.

[56] Stork MJ, Banfield LE, Gibala MJ, Ginis KAM (2017). A scoping review of the psychological responses to interval exercise: is interval exercise a viable alternative to traditional exercise?. Health Psychol Rev.

