# Supplementary material for: High-intensity interval training in children and adolescents with special educational needs: a systematic review and narrative synthesis
Source: Int J Behav Nutr Phys Act. 2023 Feb 9;20:13. doi: 10.1186/s12966-023-01421-5 (PMC9909882; doi:10.1186/s12966-023-01421-5)
Supplement: Supplementary file 1 — Additional file 1: Supplement 1. Search strategy. [file 12966_2023_1421_MOESM1_ESM.docx]

| **Terms** | **Search Strategy** |
| --- | --- |
| High intensity interval training | (“high-intensity interval training” OR “high-intensity interval exercise*” OR “high-intensity intermittent exercise” OR “high-intensity intermittent training” OR “sprint interval training” OR “sprint interval exercise” OR “interval training” OR “interval exercise” OR “HIIT” OR "high intensity training" OR "high intensity exercise*" OR "high intensity activit*" OR “vigorous training” OR "vigorous intensity exercise*" OR "vigorous activit*" OR “aerobic interval training” OR “aerobic interval exercise”) |
| Special education needs | (“SEN” OR “special educational need*” OR “physical disabilit*” OR “intellectual disabilit*” OR “visual impairment” OR “hearing impairment” OR “attention deficit/hyperactivity disorder*” OR “ attention*deficit hyperactivity disorder *” OR “ADHD” OR “autism spectrum disorder*” OR “autism or specific learning difficult*” OR “learning difficult*” OR “speech and language impairment” OR “language impairment” OR “mental illness*” OR “mental disorder*” OR “social development” OR “developmental disorder*” OR “disabilit*” OR “handicap”) |
| Children and adolescents | (“children” OR “child” OR “adolescen*” OR “youth” OR “teenager*” OR “school*aged children” OR “student*” OR “elementary school” OR “primary school” OR “junior high school” OR “middle school”) |

**Supplement 1** Search strategy
