# Supplementary material for: High-intensity interval training in children and adolescents with special educational needs: a systematic review and narrative synthesis
Source: Int J Behav Nutr Phys Act. 2023 Feb 9;20:13. doi: 10.1186/s12966-023-01421-5 (PMC9909882; doi:10.1186/s12966-023-01421-5)
Supplement: Supplementary file 2 — Additional file 2: Supplement 2. Summary of results of all included studies. [file 12966_2023_1421_MOESM2_ESM.docx]

**Supplement 2** Summary of results of all included studies

| **Study** | **Body composition** | **Physical fitness-related outcomes** | **Cardiometabolic risk biomarkers** | **Mental health/ cognitive performance** | **Adverse events/ adherence** |
| --- | --- | --- | --- | --- | --- |
| Boer et al. 2014 (31) | BMI +  Waist circumference +  Body fat % + | Cardiorespiratory fitness (cycle ergometer) +  Muscular fitness (sit-to-stand test and MRF)  Functional capacity (6MWT) + | Blood pressure +  Lipid profile +  Fasting insulin +  HOMA-IR + |  | No adverse events |
| Braaksma 2022 (36) |  | Cardiorespiratory fitness (20mSRT) +  Muscular fitness (isometric strength) +  Anaerobic performance (MPST) + |  |  | Adherence: 89% |
| Lauglo et al 2016 (37) | BMI +  Body fat % +  Fat mass 0 | Cardiorespiratory fitness (treadmill) + |  | Health-related quality of life + | No adverse events |
| Leahy et al. 2021 (30) |  | Muscular fitness (sit-to-stand test and modified push-up test) +  Functional capacity (6MWT) + |  | Mood (Feeling State questionnaire) + | No adverse events |
| Lee et al. 2019 (35) |  |  |  | Inhibitory control (CWST) + |  |
| Messler et al. 2018 (32) |  | Cardiorespiratory fitness (cycle ergometer) +  Motor skills (M-ABC-II) + |  | Health-related quality of life +  Social behavior + |  |
| Schranz et al. 2018 (33) |  | Muscular fitness (isometric strength) +  Anaerobic performance (MPST) +  Functional capacity (Timed stairs test, 6MWT, gait profile score and timed up and go test) 0 |  | Participation questionnaires + | Adherence: 88% |
| Smati et al. 2022 (28) |  | Functional capacity (6MWE, 10M-WT, 10M-FWT and 50M-ST) +  Gait efficiency + |  | Quality of life (PHSCS- 2) + | Adherence: 100% |
| Soori et al. 2020 (34) | BMI +  Fat mass + | Anaerobic performance (40-m MST) + | Lactate +  IL-13 +  IL-16 0 | CPRS + | Adherence: 100% |
| Taylor et al. 2019 (39) | BMI +  Waist circumference +  Body fat % + | Cardiorespiratory fitness (step test) +  Anaerobic performance (Wingate test) + | Resting HR 0  Blood pressure 0 | PANSS +  WHO-5 well-being index + | Adherence: ~70% |
| Torabi et al. 2018 (38) | BMI +  Fat mass + | Motor proficiency + | Adiponectin level + Insulin resistance + |  |  |
| Wymbs et al 2021 (29) |  |  |  | Mood –  Behavior –  Academic productivity 0 |  |
| Zwinkels et al. 2018 (40) | BMI 0  Waist circumference 0  Fat mass 0 | Cardiorespiratory fitness (SRT) +  Muscular fitness (standing broad jump/ 1-stroke push) +  Anaerobic performance (MPST)+ | Blood pressure +  Arterial stiffness 0  Lipid profile 0  Fasting glucose 0 |  | Adherence: 84.5%  No adverse event |

*10M-WT* 10-meter walking test, *10M-FWT* 10-meter fast walking test, *20mSRT* 20-Metre Shuttle Run Test, *50M-ST* 50-meter sprint test, *6-MWE* 6-min walking exercise, *6MWT* 6-minute walk test, *BMI* body mass index, *CPRS* Conners’ Parents Rating Scale, *CWS* Colour-Word Stroop Task, *HOMA-IR* Homeostasis model assessment of insulin resistance, *IL-13* Iinterleukin-13, *IL-16* Interleukin-16, *MPST* Muscle power sprint test, *PANSS* Positive and Negative Syndrome Scale, *M-ABC-II* Movement Assessment Battery for Children II, *MRF* Muscle fatigue resistance, *MST* Maximal shuttle run test, *PHSCS- 2* Piers-Harris Children’s Self Concept Scale-2nd edition, *SRT* Shuttle run/ride test, *VO_2peak_* Peak Oxygen Uptake

“+” denotes improvement; “0” denotes no effect; “–” denotes worsening
